# Use of hyaluronic acid injection after arthroscopic release in lateral patellar compression syndrome with degenerative cartilage changes: randomized control trial

**DOI:** 10.1186/s12891-020-03876-0

**Published:** 2021-01-06

**Authors:** Sherwan A. Hamawandi

**Affiliations:** Department of Orthopaedics, College of Medicine, Hawler Medical University, Erbil, Iraq

**Keywords:** Hyaluronic acid injection, Lateral patellar compression syndrome, Arthroscopic release, Kujala score, Visual analogue scale, Knee pain, Functional outcome

## Abstract

**Background:**

Degenerative cartilage changes can be seen, in cases of lateral patellar compression syndrome, involving the patellofemoral joint. Hyaluronic acid is a natural component of the synovial fluid and responsible for its elastic features and function of articular surfaces. The aim of this study is to show the effect of intra-articular injection of Hyaluronic acid, after arthroscopic lateral release in lateral patellar compression syndrome, on the functional outcome and knee pain in those patients with degenerative cartilage changes.

**Method:**

Ninety patients age (30–50) years with lateral patellar compression syndrome and degenerative cartilage changes were divided randomly into 2 groups. Group A was treated by arthroscopic lateral release and received intraarticular injection of Hyaluronic acid 2 weeks after surgery. Group B was treated by arthroscopic lateral release only. Both groups were assessed by Kujala score and visual analogue scale for knee pain preoperatively and re-assessed postoperatively at 3 months, 6 months, 12 months and 24 months.

**Results:**

There was significant improvement in Kujala score and Visual analogue scale post-operatively in both groups (*P*-value< 0.001) with better improvement in Kujala score in group A after intra-articular injection of Hyaluronic acid up to 2 year of follow up (*P*-value = 0.006) as well as better improvement in visual analogue score at 6 months post-operatively (*P*-value = 0.035).

**Conclusion:**

Intra-articular injection of Hyaluronic acid after arthroscopic release, in patients with lateral patellar compression syndrome and degenerative cartilage changes, can result in better improvement of knee pain and functional outcome up to 2 years of follow up.

**Trial registration:**

NCT, NCT04134611. Registered 18 October 2019 -Retrospectively registered.

## Background

Lateral patellar compression syndrome is characterized by localized dull anterior knee pain, exacerbated by activities that stress the patellofemoral joint, such as stair climbing, squatting and prolonged sitting with flexion of the knee. In such syndrome, there is increased patellofemoral joint pressure and venous engorgement of the patella and this can lead to degenerative cartilage changes [1–4].

After failure of conservative treatment for lateral patellar compression syndrome, lateral patellar retinacular release is a surgical option, which can be done arthroscopically in selected patients [5–9]. Clifton et al. [5] showed in their study that lateral patellar tilt without subluxation can benefit from lateral release in absence of grade III or grade IV changes in articular cartilage. Elkousy H [6]. showed in his study that lateral retinacular release will reduce the compressive forces on the lateral patellofemoral joint and consequently reduction of pain. Pagenstert et al. [7] used open lateral release for lateral patellar compression syndrome in their study and found better results with open lengthening that open release of lateral retinaculum. Lattermann et al. [8] showed in their systematic review of literatures that if performed in appropriate patient population, an isolated lateral retinacular release has a good chance for success. Sanchis-Alfonso et al. [9] showed that the primary indication for isolated lateral retinacular release is limited to patients with symptomatic tight lateral retinaculum and absence of patellar instability that has failed to improve with non-operative treatment.

Hyaluronic acid is a natural component of the synovial fluid and responsible for its elastic features and function of articular surfaces [10, 11]. Laurent et al. [10] showed that hyaluronan is the major component of synovial tissue and described its physiological functions like lubrication and water homeostasis. Seror et al. [11] showed that hyaluronan provides the extreme lubrication of the synovial joints via the hydration-lubrication mechanism.

Intra-articular injection of Hyaluronic acid is one of the recommendations for treatment of degenerative joint diseases [12–18]. Zhang et al. [12] showed in their recommendations that intrarticular injection of Hyaluronic acid can improve function, reduce stiffness and decrease pain with statistically significant results at 12 weeks after injection. Gadek et al. [13] confirmed in their study the effectiveness and safety of intraarticular injection of Hyaluronic acid in the treatment of knee degenerative disease. Das et al. [14] concluded that Hyaluronic acid injection can relieve pain more than placebo in knee degenerative disease. Migliore et al. [15] showed that intraarticular injection of Hyaluronan is not only symptom-modifying therapy but also a treatment which may significantly decrease deterioration of joint structure in knee osteoarthritis. Gupta et al. [16] provided in their review a mechanism-based rationale for the use of Hyaluronic acid in some diseases with special reference to osteoarthritis. Blanch et al. [17] showed that Hyaluronic acid is beneficial in osteoarthritis for the management of pain and improvement in physical function of joints. Linthoudt et al. [18] reported that intraarticular injection of Hyaluronic acid is one of the current drug recommendations for knee osteoarthritis.

On review of literatures regarding lateral patellar compression syndrome associated with degenerative cartilage changes, there is only one study; which showed the effect of arthroscopic lateral release followed by viscosupplementation for treatment of patellofemoral osteoarthritis [19]. There was no previous randomized controlled trial to show the effect of arthroscopic lateral release followed by hyaluronic acid injection for treatment of lateral patellar compression syndrome with degenerative cartilage changes so we planned our study to show the effectiveness of such method of treatment on pain relief and functional outcome with follow up for 2 years.

## Methods

### Study design

This study is a single center, double blinded, prospective, randomized, comparative, controlled trial. For all patients, the arthroscopic procedure and intra-articular injection of Hyaluronic acid were done by same orthopedic surgeon, to avoid bias related to difference in the skill of different surgeons, in a tertiary orthopedic hospital from June 2017 to March 2018 with follow up for 2 years until March 2020.

### Patients

Ninety patients age (30–50 years) were involved in this study. All these patients were diagnosed as lateral patellar compression syndrome by clinical background, MRI and proved by arthroscopic examination. Conservative treatment of quadriceps strengthening exercise and non-steroidal anti-inflammatory analgesics was failed for 6 months in these patients. All these patients were treated by arthroscopic lateral patellar release for lateral patellar compression syndrome and patellofemoral cartilage degenerative changes were found. These patients were divided randomly into two groups. Group A (45 patients) were treated by local single injection of Hyaluronic acid (MW 750 KD, Mylan company, United States) intraarticularly, 2 weeks after arthroscopy, while group B (45 patients) did not receive intraarticular injection of Hyaluronic acid, but they received intraarticular saline placebo injection. Both groups were assessed preoperatively and postoperatively at periods of 3 months, 6 months, 12 months and 24 months by using Visual analogue scale for knee pain and Kujala score for functional outcome.

### Method of randomization and blindness of the study

These 90 patients were randomly divided into two groups by entering the names of patients in excel file and by computer system the list was randomized then the patients with odd number sequences were regarded as group A and those patients with even number sequences were regarded as group B. Allocation ratio was 1:1.This study is double blinded so the patients are blinded to which method would be used for them and the doctor who assessed the patients, preoperatively and post-operatively, is blinded for which group the patients were belonged.

### Inclusion criteria

This study involved patients were diagnosed as lateral patellar compression syndrome according to all of the following criteria and all these patients had failure to conservative treatment of quadriceps strengthening physiotherapy and non-steroidal anti-inflammatory drugs for 6 months [6, 20].

1. Patients, were included in this study, had most pain and tenderness on the lateral region of the patella with abnormal patellar tilt test and abnormal medial patellar glide test. When the examiner could not lift the patella from the lateral femoral condyle with knee in extension, this means abnormal patellar tilt test. When the patella could not be shifted by one or more quadrants medially with 10 degrees of knee flexion, this was regarded as abnormal medial patellar glide test [7, 21].
2. To be included in this study, patients should have patellar translation, abnormal patellar tilt and early stage I and stage II degenerative cartilage changes in the lateral part of patellofemoral joint. Patellar translation is defined as more than 2 mm distance between a line drown at the medial edge of the patella and another parallel line drown at the anterior border of the medial femoral condyle on the axial MRI image [22]. Abnormal patellar tilt is defined as less than 8 degrees of patellofemoral angle measured at midpoint of the patella on sagittal MRI image [22]. Stage I degenerative cartilage change is defined as slight elevation of surface signal on MRI image while stage II is defined as chondral fissure involved less than 50% of articular cartilage thickness on MRI image [23].
3. Arthroscopic assessment was done in all patients to evaluate the dynamic contact between the patella and lateral femoral condyle during knee flexion and extension movements as well as to evaluate the degenerative cartilage lesion of the patellofemoral joint. Patients with Outerbridge grade I and grade II were included in this study. Outbridge grade I chondral lesion is defined as swelling and softening of articular surface that can be assessed with a probe during arthroscopy. Outbridge grade II lesion is defined as a chondral lesion with partial thickness defect of less than half inch diameter [24].

### Exclusion criteria

Exclusion criteria included:

1. Patient’s age more than 50 years old.
2. Diabetes Mellitus
3. Smoking
4. Osteoarthritis of tibiofemoral component of the knee joint [7].
5. Previous surgery or infection of the knee joint.
6. Patella alta when Caton-Deschamps index is more than 1.3
7. Instability of the patella which is defined as medial or lateral glide test of three quadrants or more.
8. History of dislocation of the patella.
9. Generalized ligamentous laxity which is defined by Beighton’s criteria [25].
10. Rotational malalignment of lower limbs as pathological femoral anteversion or tibial torsion which are assessed clinically by using Steheli’s test [7, 26].
11. Frontal malalignment with Q-angle more than 20 degrees [7, 27].
12. Chondral lesions grade III and grade IV. Degenerative cartilage changes of grade III chondropathy is defined as fissuring of articular cartilage involving more than 50% thickness on MRI images and more than half inch fissures seen during arthroscopic assessment. Grade IV chondropathy is defined as fissure in the articular cartilage that reaches to the subchondral bone on MRI images and cartilage lesion with exposure of subchondral bone during arthroscopic assessment [23, 24].
13. Dejour’s types II, III and IV dysplastic femoral trochleae [7, 28].

### Follow up and outcome measures

All these patients were assessed for primary outcome measure of Kujala score and secondary outcome measures which involved Visual analogue scale for knee pain and postoperative complications of bleeding, infection, medial patellar instability and recurrence of knee pain for a period of 2 years follow up.

All patients were assessed by Kujala score for functional outcome and VAS for knee pain preoperatively and postoperatively at 3 months, 6 months, 12 months and 24 months.

There was no loss of patients during the period of follow up for 2 years.

### Intervention

Under general or spinal anaesthesia, patient was on supine position with pneumatic thigh tourniquet on the side of operation and attatched to a leg holder. Knee arthroscopy was done through standard anterolateral and anteromedial portals. Examination of all compartments of the knee were assessed and observing the patellar movement on the trochlear groove during flexion and extension of the knee to assess the contact between the patella and lateral part of trochlear groove. Assessment of the patellofemoral joint for the chondral lesion. Lateral patellar retinacular release was done by back-knife and electrocoutary with continous monitoring of the patellar movement in the trochlear groove and the patellar contact with the lateral femoral condyle during knee movement in flexion and extension. Hemostasis was secured and drain was not used.

### Post-operative care

All patients were encouraged to do quadriceps exercise and gradual weight bearing as early as possible with immediately full active range of movement and no knee brace was used. Low-molecular weight heparin was prescribed for 2 weeks after surgery. After 2 weeks, single intraarticular injection of hyaluronic acid (60 mg) was done in group A and single intraarticular saline placebo injection in group B. All patients were assessed by Visual analogue score for knee pain and Kujala score at 3 months, 6 months 12 months and 24 months postoperatively.

### Post-operative complications

1. Hemarthrosis occurred in 5 patients; 3 of them in group A and 2 patients in group B. All of them were treated by aspiration and firm bandage.
2. One case got DVT in group B and was treated by anticoagulant medication.
3. No case of infection
4. No case of recurrence for 2 year of follow up

Recurrence is defined as painful Passive Patellar Tilt test with Medial Patellar Glide test of less than 1 quadrant of patellar width according to Kolowich et al. [7, 29]

5/ No case of medial patellar instability.

Medial patellar instability is defined as medial patellar translation of three or more quadrants of patellar width on Medial Patellar Glide test with positive Gravity Subluxation test according to Nonweiler and DeLee [7, 30].

### Data analysis

Statistical analysis was carried out using SPSS version 23 (IBM, Armonk, NY, USA). Categorical variables were presented as frequencies and percentages. Continuous variables were presented as (Means ± SD). Student t-test was used to compare means between two groups. Paired t-test was used to compare two paired readings. Chi-square test was used to find the association between categorical variables. A *p-*value of ≤0.05 was considered as significant.

## Results

### Demographic data

In current study, 90 patients were involved. The mean age of patients was (41.13 ± 6.10). The younger age was 30 years and the older one was 50 years. There were 23 males who represent (25.6%) and 67 females who represent (74.4%). The mean age of group A was 42.13 and the mean age of group B was 40.13. In group A, males were 12 and females were 33 while in group B, males were 11 and females were 34. There were no significant differences between means of age between these two groups (*P* = 0.121), as well as there was no significant association between gender and study groups, (*P* = 0.809).

### Primary outcome measure: (Kujala score)

In group A, there were significant differences (*P*-value < 0.001) in the mean of Kujala score between pre-operative and post-operative assessments in four time periods (3 months, 6 months, 12 months and 24 months) as shown in Table 1.

**Table 1:** The mean differences of Kujala score between pre-operative and post-operative assessments in four time periods for group A

Study variables	Periods of assessment	N	Mean	SD	Paired t-test	***P***-value
Kujala score	Kujala score preoperatively	45	40.60	6.03	**− 36.57**	**< 0.001٭**
	Kujala score 3 months postoperatively	45	83.28	4.61		
	Kujala score preoperatively	45	40.60	6.03	**−42.57**	**< 0.001٭**
	Kujala score 6 months postoperatively	45	84.62	3.09		
	Kujala score preoperatively	45	40.60	6.03	**−46.96**	**< 0.001٭**
	Kujala score 1 year postoperatively	45	86.08	1.59		
	Kujala score preoperatively	45	40.60	6.03	**− 48.18**	**< 0.001٭**
	Kujala score 2 year postoperatively	45	87.11	1.97		

*means statistically significant

In group B, there were significant differences (*P*-value< 0.001) in the mean of Kujala score between pre-operative and post-operative assessments in four time periods (3 months, 6 months, 12 months and 24 months) as shown in Table 2.

**Table 2:** The mean differences of Kujala score between pre-operative and post-operative assessments in four time periods for group B

Study variables	Periods of assessment	N	Mean	SD	Paired t-test	***P***-value
Kujala score	Kujala score preoperatively	45	40.64	5.83	**−34.63**	**< 0.001٭**
	Kujala score 3 months postoperatively	45	76.35	3.48		
	Kujala score preoperatively	45	40.64	5.83	**− 37.46**	**< 0.001٭**
	Kujala score 6 months postoperatively	45	77.93	3.10		
	Kujala score preoperatively	45	40.64	5.83	**− 44.39**	**< 0.001٭**
	Kujala score 1 year postoperatively	45	84.82	3.00		
	Kujala score preoperatively	45	40.64	5.83	**−44.99**	**< 0.001٭**
	Kujala score 2 year postoperatively	45	85.822	2.33		

*means statistically significant

**Fig. 1:**
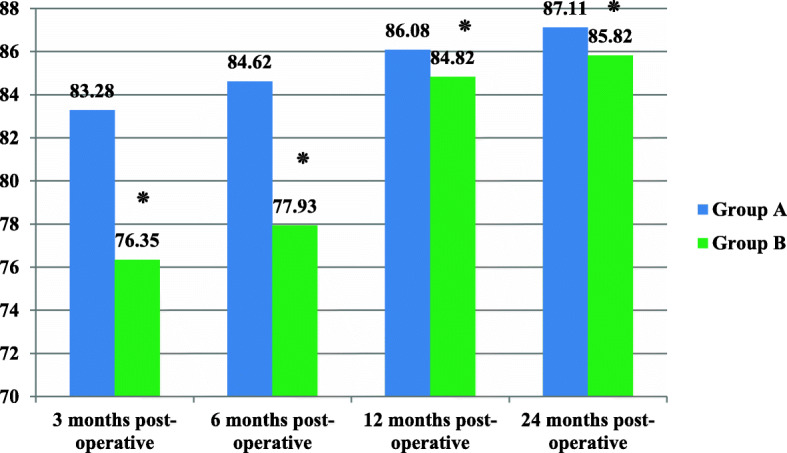
The mean differences of post-operative Kujala score between study groups

Figure 1 showed the mean differences of post-operative Kujala score between study groups including (group A and group B) in four periods of assessments. There were significant differences between means of Kujala score for knee pain between these two groups after 3 months postoperatively (t = 8.048, *P* = < 0.001٭), and after 6 months (t = 10.225, *P* = < 0.001٭), and after 1 year (t = 2.50, *P* = 0.015٭) and 2 years(t = 2.822, *P* = 0.006٭) of operation.

### Secondary outcome measures

#### Visual analogue scale for knee pain

In group A, there were significant differences (*P*-value< 0.001) in the mean of VAS score for knee pain between pre-operative and post-operative assessments in four time periods (3 months, 6 months, 12 months and 24 months) was shown in Table 3.

**Table 3:** The mean differences of VAS score for knee joint between pre-operative and post-operative assessments in four time periods for group A

Study variables	Periods of assessment	N	Mean	SD	Paired t-test	***P***-value
VAS score	VAS score preoperatively	45	8.44	0.50	**25.02**	**< 0.001٭**
	VAS score 3 months postoperatively	45	2.06	1.49		
	VAS score preoperatively	45	8.44	0.50	**29.83**	**< 0.001٭**
	VAS score 6 months postoperatively	45	1.31	1.39		
	VAS score preoperatively	45	8.44	0.50	**43.09**	**< 0.001٭**
	VAS score 1 year postoperatively	45	0.80	0.99		
	VAS score preoperatively	45	8.44	0.50	**50.99**	**< 0.001٭**
	VAS score 2 year postoperatively	45	0.53	0.89		

*means statistically significant

In group B, there were significant differences (*P*-value< 0.001) in the mean of VAS score for knee pain between pre-operative and post-operative assessments in four time periods (3 months, 6 months, 12 months and 24 months) was shown in Table 4.

**Table 4:** The mean differences of VAS score for knee pain between pre-operative and post-operative assessments in four time periods for group B

Study variables	Periods of assessment	N	Mean	SD	Paired t-test	***P***-value
VAS score	VAS score preoperatively	45	8.31	0.46	**23.21**	**< 0.001٭**
	VAS score 3 months postoperatively	45	2.48	1.51		
	VAS score preoperatively	45	8.31	0.46	**23.93**	**< 0.001٭**
	VAS score 6 months postoperatively	45	2.00	1.65		
	VAS score preoperatively	45	8.31	0.46	**32.86**	**< 0.001٭**
	VAS score 1 year postoperatively	45	0.88	1.30		
	VAS score preoperatively	45	8.31	0.46	**45.89**	**< 0.001٭**
	VAS score 2 year postoperatively	45	0.51	0.92		

*means statistically significant

**Fig. 2:**
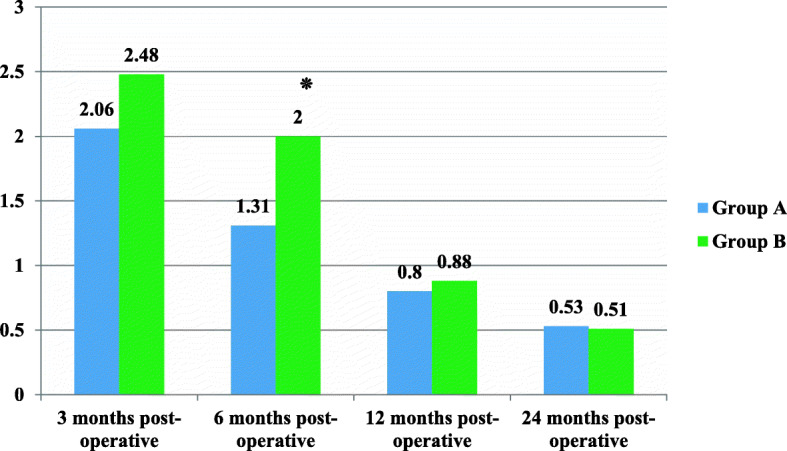
The mean differences of post-operative VAS score for knee pain between study groups

Figure 2 showed the mean differences of post-operative VAS score for knee joint between study groups including (group A and Group B) in four periods of assessments. There were significant differences between means of VAS score for knee joint between these two groups after 6 months postoperatively (t = − 2.138, *P* = 0.035٭), while non-significant differences between two groups after 3 months(t = − 1.328, *P* = 0.187), 1 year (t = − 0.365, *P* = 0.716) and 2 years(t = 0.116, *P* = 0.908) of operation.

#### Post-operative complications

There were 3 patients in group A and 2 patients in group B suffered from post-operative hemarthrosis and were treated by aspiration and firm bandaging without need for another arthroscopy.

There was one patient in group B suffered from DVT of leg on side of operation and was treated with anticoagulant after consultation with hematologist.

Fortunately, I had no infection, recurrence or patellar instability with 2 years of follow up.

## Discussion

On review of previous literatures, only one article can be found to study using of intra-articular injection of Hyaluronic acid after arthroscopic lateral release in lateral patellar compression syndrome, Fosca et al. [19] study was retrospective study involved 25 patients and showed that mean of Kujala score improved from 45.8 before surgery to 82.7 after treatment and Visual analogue scale improved of 68.6% from preoperative assessment, while current study was randomized controlled trial involved 90 patients and showed significant improvement in Kujala score and VAS score after arthroscopic lateral release and intraarticular injection of Hyaluronic acid.

When I compared current study with other studies that exclusively analyzed lateral patellar release or viscosupplementation, I can find the following important points:

1. Chen et al. [31] reported that arthroscopic lateral patellar retinacular release can effectively improve function and symptoms of patellofemoral joint in patients with lateral patellar compression syndrome. In current study, there was improvement after lateral release with better results after intraarticular injection of Hyaluronic acid as those patients, who are involved in current study, had degenerative changes in patellofemoral joint.
2. Fithian et al. [32] showed in their scientific survey among experienced knee surgeons that lateral release should be done with prior planning in the form of objective clinical indications and this was considered in current study through rigorous inclusion and exclusion criteria.
3. Fu et al. [33] concluded that lateral release can provide dramatic relief of anterior knee pain in selected patients. In current study, carefully planned eligibility criteria were considered.
4. Astur et al. [34] found that intraarticular Hyaluronic acid injection can provide less pain and better function in those patients with patellar chondropathy of grades II and III. In current study, there was better results regarding pain relief and knee function after Hyaluronic acid injection in patients with degenerative changes of patellofemoral joint after lateral release.

### Limitations of the study

1. The duration from the onset of symptoms to time of arthroscopy was not analyzed in this study.
2. Long term follow up is recommended for future studies.

## Conclusion

Intra-articular injection of Hyaluronic acid after arthroscopic release, in patients with lateral patellar compression syndrome and degenerative cartilage changes, can result in better improvement of knee pain and functional outcome up to 2 years of follow up.

## Data Availability

The datasets used and analyzed during the current study are available from the corresponding author on reasonable request.

## Abbreviations

VAS: Visual analogue scale
MRI: Magnetic resonance imaging
DVT: Deep venous thrombosis
